# Effects of Coated Sodium Selenite Supplementation on the Milk Yield, Apparent Digestibility, Rumen Fermentation, Blood Biochemical Parameters and Antioxidant Parameters in Dairy Buffaloes

**DOI:** 10.3390/ani15192767

**Published:** 2025-09-23

**Authors:** Li Tan, Yuqi Zhao, Jiajin Sun, Chun Bai, He Du, Xinyu Yan, Gang Guo, Lei Chen, Qiang Liu, Cong Wang, Wenjie Huo

**Affiliations:** College of Animal Science, Shanxi Agricultural University, Jinzhong 030801, China; tanli152@163.com (L.T.); 18235810139@163.com (Y.Z.); jiajinsun@stu.sxau.edu.cn (J.S.); 18234876101@163.com (C.B.); 15642147811@163.com (H.D.); 18735806412@163.com (X.Y.); guosteel1984@163.com (G.G.); cl1016zj@126.com (L.C.); liuqiangabc@163.com (Q.L.); wangdx0321@163.com (C.W.)

**Keywords:** blood biochemical parameters buffalo, coated sodium selenite, lactation performance, rumen fermentation

## Abstract

Coated sodium selenite (CSS) is a rumen-protected selenium supplement that can improve selenium status and lactation performance in buffalo. This study investigated the effects of CSS supplementation on milk yield, rumen fermentation, digestibility, blood biochemical parameters, and antioxidant capacity in 28 dairy hybrid buffaloes (Murrah × local breed; milk yield = 5.96 ± 0.21 kg/d; parity = 2.96 ± 0.15, mean ± SD). The buffaloes were randomly allocated into four groups: control (basal diet), low CSS (LCSS, basal diet + 0.1 mg/kg CSS), medium CSS (MCSS, basal diet + 0.15 mg/kg CSS), and high CSS (HCSS, basal diet + 0.2 mg/kg CSS). The trial included a 7-day adaptation period followed by a 60-day experimental period. Compared with the control group, the LCSS group showed significant increases in rumen acetic acid, propionic acid, and total volatile fatty acid contents; milk yield, milk fat percentage, and 4% standard milk yield; neutral detergent fiber digestibility; and antioxidant capacity. These results demonstrate that supplementing 0.1 mg/kg CSS improves rumen fermentation efficiency and cellulose digestibility, thereby enhancing the antioxidant capacity and lactation performance of dairy buffaloes.

## Simple Summary

Selenium is one of the essential trace elements. It plays a vital role in animal health and reproduction, primarily through its involvement in antioxidant defense systems. Exploring the optimal level and form of selenium addition can help improve the nutritional management level of buffalo farmers and avoid common mineral imbalances in buffalo production. It is of great significance to promote the efficiency improvement and sustainable development of the buffalo industry. This study used coated sodium selenite as a selenium supplement to investigate the effects of different addition levels on the production performance of buffaloes. The results indicate that the addition of coated sodium selenite can improve the lactation performance, digestive function, and antioxidant capacity of buffaloes. Therefore, this study shows that the addition of coated sodium selenite can improve the milk yield of buffaloes, which provides a valuable reference for feed nutrition regulation in the buffalo industry.

## 1. Introduction

According to the Food and Agriculture Organization (FAO), the buffalo (*Bubalus bubalis*) is the world’s second largest source of milk and plays a strategic role in the world economy and society. In order to improve the profitability of dairy buffaloes and expand the market of the buffalo milk industry, it is necessary to analyze and solve the current problems of low per capita milk production, reproductive inefficiency, and slow growth of buffaloes due to inadequate nutrient supply to buffaloes [1,2]. Among these challenges, imbalances in dietary mineral supplementation are particularly common due to the lack of precise nutritional guidelines, a situation exacerbated by the current reliance on cow data for buffalo farming. Therefore, exploring the optimal mineral levels and providing better mineral nutritional management will not only avoid the imbalance in dietary mineral supplementation that is common in buffalo production [3] and improve the efficiency of buffalo production to some extent, but also help the buffalo industry to achieve a healthier and more sustainable development.

Selenium (Se), as one of the essential trace minerals, plays a central role in enhancing the antioxidant defense mechanisms of organisms. Adding Se to the diet increases the antioxidant capacity of animals, reduces free radical damage, and improves their immune function [4,5,6]. In addition, the antioxidant capacity of Se has been shown to improve rumen fermentation and increase digestibility. Čobanová et al. [7] reported that with increasing Se content, Se concentration and glutathione peroxidase (GSH-Px) activity in rumen microorganisms and duodenal mucosa increased, resulting in improved nutrient digestion and animal performance. Zhang et al. [8] found that the inclusion of 0.3 mg/kg Se in the diet of Holstein cows improved rumen fermentation, increased total volatile fatty acid (VFA), and decreased rumen pH, ammoniacal nitrogen content, and acetic acid/propionic acid ratio, which improved the antioxidant status of microbial cells and the rumen environment and promoted microbial growth and enzyme activity. Furthermore, selenium is integral to mammalian reproductive physiology, influencing crucial processes such as spermatogenesis and embryonic development [9,10]. It is important to note that while the beneficial effects of Se supplementation are well-documented in dairy cows, specific nutritional data for buffaloes remain relatively limited.

The major forms of Se currently available in diets are inorganic and organic Se. Inorganic Se added to the ration is partially reduced to insoluble elemental Se in the rumen by the metabolic action of rumen microorganisms, resulting in reduced absorption. In contrast, most of the Se in organic Se leaves the rumen as selenoamino acids and is absorbed in the duodenum with high utilization [11,12,13]. Gresakova et al. [14] also reported that the addition of Se yeast to the diet resulted in higher Se absorption in the digestive tract of lambs compared to the addition of selenite to the diet. However, organic Se sources are often more expensive than inorganic ones. However, organic Se sources are often more expensive than inorganic ones. To bridge this gap between cost and efficacy, rumen-protected forms of inorganic Se have been developed. Coated sodium selenite (CSS) is a rumen-protected Se supplement with a utilization rate comparable to that of organic Se. The addition of CSS was more efficacious than sodium selenite in promoting lactation performance in dairy cows [8]. Du et al. [15] found that the addition of CSS increased milk yield, total VFA, and total bacterial, protozoan, and fungal counts in dairy cows. Furthermore, by using different coating aids and different processing techniques, CSS can meet a wider range of needs and is an efficacious method of Se supplementation.

To date, no research has been conducted to ascertain the effects of CSS supplementation on buffalo. Therefore, the research team devised a bespoke CSS to circumvent the metabolic effects of rumen microbes. We hypothesized that CSS supplementation would improve lactation performance, nutrient digestibility, ruminal fermentation, and antioxidant status in dairy buffaloes. The aim of this experiment was to investigate the effect of CSS on milk yield, milk quality, nutrient digestion, rumen fermentation, blood biochemical parameters, and antioxidant parameters of dairy buffaloes. By exploring the optimum mineral level and providing better mineral nutrition management, the milk yield of buffaloes will be increased, and lactation potential will be improved.

## 2. Materials and Methods

The present study was approved by the Animal Health and Care Committee of the Shanxi Agricultural University (Shanxi, China) and conducted according to the Guidelines for the Experimental Animal Welfare of Ministry of Science Technology of China (approval code: SXAU-EAW-2022C.RD.010025174). The facility was inspected and approved by the Institutional Animal Care and Use Committee. The buffaloes were accommodated in a barn that was both naturally ventilated and mechanically ventilated, thereby providing them with sufficient space for exercise and rest. The buffaloes were fed ad libitum, watered ad libitum.

### 2.1. Animals, Experimental Design and Management

The experiment was carried out at a Fangchenggang dairy buffalo producing farm (Fangchenggang, China). The animals were a hybrid of the Murrah buffalo and local buffalo breeds. Twenty-eight dairy buffaloes (milk yield = 5.96 ± 0.21 kg; parity = 2.96 ± 0.15, mean ± SD) were selected and randomly assigned to one of four groups (*n* = 7) based on random sequences generated by the RAND function in Excel. The groups were designated as control (without CSS supplementation), low CSS (LCSS), medium CSS (MCSS), and high CSS (HCSS), as follows:

Control group: basal diet (Con);
Treatment 1: basal diet + CSS 0.10 mg/kg (LCSS);
Treatment 2: basal diet + CSS 0.15 mg/kg (MCSS);
Treatment 3: basal diet + CSS 0.20 mg/kg (HCSS).

This experiment is based on the species differences between dairy buffaloes and Holstein cows, as well as the objective situation of slow growth and development and low milk production of dairy buffaloes. Referring to the dietary formula of local enterprises and the recommended Se content (0.3 mg/kg) for dairy cows in the NRC [16], this experiment designed diets with different Se contents based on the recommended Se content (0.1 mg/kg) higher than the NRC Beef Cattle Standard. The CSS employed in this experiment was developed by the research group and the specific production process was described by Wang et al. [8]. The formulation consisted of 300 g/kg SS premix (5 g/kg Se), 370 g/kg hydrogenated fat (C16:0:C18:0 = 2:1), 170 g bentonite powder, and 160 g/kg calcium stearate. All ingredients except fat were first mixed uniformly. The hydrogenated fat was heated to 80 °C, then incorporated into the mixed ingredients. The mixture was subsequently pelletized into 1.00–1.25 cm particles using a rotating pelletizer (HJ-400-S; Chongqing Rongkai Machinery Manufacturing Co., Ltd., Chongqing, China). The release rate of Se from CSS in the rumen is 28%, and the release rate of Se in the small intestine is 58%.

The adaptation period lasted for 7 d, followed by a 60 d experimental period. The basal diet was formulated according to the internal feed specifications of Guangxi Royal Dairy Co., Ltd. (Nanning, China). It was provided as a total mixed ration (TMR), consisting of corn silage and mixed concentrates on a dry matter (DM) basis (Table 1). The TMR was fed twice daily, while the CSS was supplemented individually at 8:00 a.m. each day.

### 2.2. Sampling and Data Collection

The feed provided to buffaloes and the refusals were weighed and collected individually per animal daily throughout the experimental period, and the dry matter intake (DMI) was analyzed.

The dairy farm used a tandem milking parlor with a milking machine. Milk samples were collected at 6:30 a.m. and 6:30 p.m. every 10 days during the experiment, respectively. The collected milk samples were mixed according to the weighted average of milk yield.

During the experimental period, feed and feed leftover samples were collected every 10 days, with approximately 2 kg collected each time, and stored at −20 °C. After the experimental period, the feed and residue samples were mixed in proportion, dried in an oven at 65 °C for 3 days, and dried to constant weight after 1 day of moisture regain. The samples were then crushed, sieved through a 1 mm sieve, sealed, and stored for later analysis.

Four days before the end of the experiment, rectal feces were collected. Fecal samples were collected at 06:00, 12:00 and 18:00 every day. About 200 g of fecal samples were collected each time and stored at −20 °C. The fecal samples collected within 4 days were mixed evenly with each buffalo as a unit, and 500 g fecal samples were taken out, and then kept at −20 °C. After the experiment, 10% tartaric acid solution was added to the samples at a ratio of 1/4 of the fresh feces weight, followed by drying in an oven at 65 °C to constant weight and determination of the initial moisture content. After that, the sample was crushed and passed through a 1 mm sieve, and the crushed sample was sealed for subsequent determination of nutrients in feces.

Rumen fluid was collected 3 h after daily feeding for 3 days prior to the end of the experiment. Rumen fluid was collected from the oral cavity of dairy buffaloes by the negative pressure method using a gastric tube. The first 30 mL was discarded to avoid saliva contamination, and a total of 50 mL of rumen fluid was collected from each buffalo. The pH of the rumen fluid was immediately measured using a PSH-3C acidimeter. A 10 mL sample was added to a vial containing 2 mL of 25% (*w*/*v*) meta-phosphoric acid for later volatile fatty acid (VFA) determination, and the sample was then frozen and stored at −20 °C for subsequent determination of ammonia nitrogen and VFA.

On the last day of the study, vacuum blood collection tubes with no additive were used to collect 10 mL of blood from dairy buffaloes via the jugular vein at approximately 10:00 a.m. Serum was extracted by centrifugation at 3000× *g* for 10 min and then frozen at −20 °C for determination of blood biochemical parameters and antioxidant parameters.

### 2.3. Chemical Analyses

The milk production was recorded daily by the dairy department of the dairy farm, and the milk sample is collected for one day to obtain the milk production data. The routine milk data was measured by Guangxi Huangshi Dairy Co., Ltd. in Nanning, Guangxi Zhuang Autonomous Region, China.

The pulverized TMR and fecal samples were required to determine the amount of dry matter (DM), crude protein (CP), crude fat (EE), neutral detergent fiber (NDF), and acid detergent fiber (ADF) contained therein. The quantities of DM (method 934.01), ether extract (EE; method 973.18), and crude ash (method 942.05) in the TMR and fecal samples were determined in accordance with the AOAC method [17]. Organic matter (OM) content was calculated as the difference between DM and crude ash content. NDF content was determined according to the following equation Van Soest et al. [18]. ADF content was analyzed according to the method described by AOAC (method 973.18) [17].

Total intestinal apparent digestibility was calculated by measuring the acid insoluble ash (AIA) content in the diets and in the feces, using AIA as an internal standard [19].

$$\mathrm{DM}\;\mathrm{digestibility}=100\times [1-\frac{AIA_{\left(\mathrm{feed}\right)}}{AIA_{\left(\mathrm{feces}\right)}}]\;$$

$$Nutrient\;digestibility=100\times [1-\frac{AIA_{\left(\mathrm{feed}\right)}}{AIA_{\left(\mathrm{feces}\right)}}\times \frac{Nutrient_{\left(\mathrm{feces}\right)}}{Nutrient_{\left(\mathrm{diet}\right)}}]$$

The rumen ammonia nitrogen concentration was determined by a colorimetric method, and after measuring the absorbance values of the samples and samples using a UV-visible spectrophotometer, the concentration of ammonia nitrogen in the rumen fluid was calculated based on the standard curve constructed from the concentration of the samples and absorbance values. VFA concentrations were analyzed by gas chromatography (GC-7890; Agilent Technology, Beijing, China).

Serum total protein (TP), albumin (ALB), total cholesterol (TC), triglyceride (TG), blood urea nitrogen (BUN), alanine aminotransferase (ALT), aspartate aminotransferase (AST), malondialdehyde (MDA), superoxide dismutase (SOD), catalase (CAT), total antioxidant capacity (T-AOC) and GSH-PX were analyzed using kits (Nanjing Jiancheng Bioengineering Institute, Nanjing, China).

### 2.4. Statistical Analysis

Fat-corrected milk (FCM) was calculated according to the NRC, where FCM = 0.4 × milk (kg/d) + 15 × fat (kg/d). The data obtained were analyzed using SPSS (Version 25.0, SPSS Inc., Chicago, IL, USA). Milk yield and milk composition were analyzed using the Mixed Model for Repeated Measures.

Y_ijkl_ = μ + C_i_ + S_j_ + T_k_ + (TS)_jk_ + R_ijkl_ + ε_ijkl_,

where Y_ijkl_ = dependent variable, μ = the overall mean, C_i_ = fixed effects of block, S_j_ = fixed effects of treatments, T_k_ = fixed effect of time, (TS)_jk_ = the treatment × time interaction, R_ijkl_ = the random effects of buffaloes and ε_ijkl_ = the residual error.

Data for apparent digestibility, rumen fermentation parameters, blood metabolites, and antioxidant parameters were analyzed by one-way ANOVA. Differences among treatment groups were compared using Duncan’s multiple range test. The significance level was set at *p* < 0.05, and trends were defined as 0.05 < *p* < 0.10.

## 3. Results

### 3.1. Lactation Performance

The results of Lactation performance are shown in Table 2. The addition of CSS significantly increased DMI (*p* = 0.001), the DMI was found to be significantly higher (*p* < 0.05) in the LCSS group than in the Con, MCSS and HCSS groups. The addition of CSS significantly increased FCM (*p* = 0.001). The milk yield and the FCM were found to be significantly higher (*p* < 0.05) in the LCSS group than in the Con and HCSS groups. The addition of CSS significantly increased fat (*p* = 0.020). The fat, lactose and total solid were found to be significantly higher in the LCSS group than other groups. Conversely, other milk compositions were not affected by the increase in CSS addition (*p* > 0.05). The addition of CSS significantly increased feed conversion ratio (*p* = 0.001).

### 3.2. Apparent Digestibility

The results of apparent digestibility are shown in Table 3. The NDF digestibility increased significantly (*p* = 0.003) following CSS addition. The addition of CSS at a low dose (0.10 mg/kg) resulted in a higher digestibility of NDF in dairy buffaloes compared to the Con group (*p* < 0.05). There was a tendency for ADF digestibility to increase with CSS supplementation (*p* = 0.087). Conversely, the digestibility of DM, OM, CP, EE and starch were not affected by the increase in CSS addition (*p* > 0.05).

### 3.3. Rumen Fermentation

The results of rumen fermentation are shown in Table 4. With increasing supplementation of CSS, ruminal pH, acetate, propionate and isobutyrate and total VFA concentration increased significantly (*p* < 0.05) and ammonia N decreased significantly. The ammonia N, butyrate, isovalerate and valerate were not affected by the increase in CSS addition (*p* > 0.05).

### 3.4. Blood Biochemical Parameters

The results of blood biochemical parameters are shown in Table 5. The TP decreased significantly (*p* = 0.001) following CSS addition. Conversely, the blood concentrations of ALB, BUN, TC, TG, ALT and AST were not affected by the increase in CSS addition (*p* > 0.05).

### 3.5. Antioxidant Parameters

The results of antioxidant parameters are shown in Table 6. The GSH-Px (*p* = 0.011) and T-AOC (*p* = 0.002) increased significantly following CSS addition. The MDA increased significantly (*p* = 0.043) following CSS addition Conversely, the CAT and SOD were not affected by the increase in CSS addition (*p* > 0.05).

## 4. Discussion

### 4.1. Lactation Performance

We found that the addition of CSS resulted in higher milk yield and FCM. Compared with other groups, LCSS group showed significant advantages in milk yield, milk fat yield and total solid yield. This is in agreement with the results of Ullah et al. [20] that the addition of yeast-based Se resulted in higher milk yield in dairy cows. This can be attributed to the positive effects of adding CSS on fiber digestibility and antioxidant parameters. Adding CSS can improve NDF digestibility by increasing cellulase activity, thereby increasing rumen VFA concentration and providing a basic substrate for the entire milk production process. Furthermore, the metabolic activity of lactating mammary glands generates large amounts of reactive oxygen species (ROS), which can damage epithelial cells and impair the function of lactation-related enzymes. Se supplementation alleviates this oxidative stress. The LCSS group exhibited significantly higher GSH-Px activity and T-AOC compared to the control group. This upregulation of antioxidant defenses helps to preserve mammary epithelial cell integrity and maintain the expression of genes and proteins essential for breast development [21]. Consequently, it directly enhances the synthesis of milk fat and total solids. The true protein content did not change significantly. This was likely because milk protein synthesis is primarily limited by dietary amino acid supply and hepatic partitioning, and selenium supplementation did not enhance protein digestibility. However, by increasing overall milk yield, CSS supplementation increased the total yield of true protein.

The effects of Se supplementation on milk composition reported in the literature vary considerably. Zhang et al. [8] observed that milk composition showed a linear increase with CSS addition. Conversely, Sun et al. [13] observed that the supplementation of hydroxy-selenomethionine in Holstein dairy cows demonstrated a tendency to reduce fat percentage in comparison with sodium selenite. It is reasonable to hypothesize that the observed discrepancies in outcomes may be attributed to differences in dietary composition, Se dose, and the lactation stage [22].

### 4.2. Apparent Digestibility

The increase in the digestibility of NDF and ADF following the addition of low doses of CSS in the present study is consistent with the findings of Zhang et al. [8] regarding the digestibility of NDF in dairy cows. In contrast, results indicate that high doses of selenium may not improve digestibility and could even have adverse effects. Zhang et al. [8] showed that the addition of CSS to the diet could significantly improve the digestibility of NDF and ADF in dairy cows, and further pointed out that this addition could enhance cellulase activity. The increase of cellulase activity was closely related to the increase of the number of anaerobic fungi and cellulose decomposing bacteria in the rumen. These results explained the increase in NDF and ADF digestibility and acetate concentration. However, when the addition of CSS exceeded 0.3 mg Se/kg DM, the rumen microbial enzyme activity showed a downward trend. These findings confirmed that excessive CSS addition was not conducive to improving rumen microbial enzyme activity, ultimately leading to the decline in NDF and ADF digestibility. The above differences may be attributed to the dose-dependent promotion effect of CSS on microorganisms.

This dose-dependent effect is consistent with findings from studies on other forms of selenium. For instance, the digestibility of DM, OM, CP, NDF and ADF was observed to increase quadratically by Shi et al. [23] when sheep were fed the basal diet supplemented with 0, 0.3, 3 and 6 g nanoselenium/kg DM. Furthermore, Del Razo-Rodríguez et al. [24] reported that the addition of Se to 70% grain rations increased the ruminal digestibility of OM and NDF, while the same level of Se added to 50% grain rations decreased the ruminal digestibility values of OM and NDF, and that Se levels were negatively correlated with ruminal digestibility.

In conclusion, our results showed that the addition of 0.1 mg/kg CSS has been observed to facilitate nutrient digestion in buffalo.

### 4.3. Rumen Fermentation

A number of factors influence pH in ruminants, including rumination, organic acid production, composition and diversity of the rumen bacterial community, and the type and composition of the diet [25,26]. Previous studies have shown that the addition of Se at appropriate doses to ruminant diets can positively affect rumen fermentation in a number of ways; the addition of Se can increase the antioxidant status of the rumen microbiome, favor the colonization of bacteria with high Se intake, high fiber digestibility and antioxidant activity, which can result in an increase in microbial proliferation and rumen function and an increase in rumen microbial fermentation rate [8,22,27]. For example, different concentrations of sodium selenite increased rumen activities of xylanase, protease, α-amylase, pectinase, cellulase, and carboxymethyl cellulase [15,28], which resulted in improved fiber digestibility, higher nutrient utilization of the diet, and higher total VFA concentration [13,15,29]. However, excessive selenium supplementation may inhibit microbial enzyme activity and reduce fermentative efficiency, ultimately diminishing VFA production and negatively impacting rumen function [8].

Therefore, the increase in NDF digestibility observed after the addition of 0.1 mg/kg CSS in this experiment is most likely due to the enhanced antioxidant capacity of buffalo. The increase in antioxidant capacity reduces the negative effects of oxidative stress on microbial communities, optimising the activity and metabolic function of rumen microorganisms and ultimately leading to an increase in NDF digestibility. Moreover, dietary supplementation of low-dose CSS was found to significantly increase rumen acetic acid, propionic acid, and total VFA content in dairy buffaloes in this study. And acetate produced by rumen fermentation is precursors for milk fatty acid synthesis. Therefore, the addition of CSS increased rumen fermentation to produce rumen acetate and propionate, which ultimately led to an increase in milk fat and FCM production. The ruminal ammonia nitrogen concentration was observed to decreased with increasing inclusion of Se, which was attributed to synthesis of microbial proteins by rumen microorganisms using the carbon skeleton and ammonia nitrogen.

The elevated ruminal pH observed under high selenium supplementation likely resulted from inhibited microbial fermentation, as evidenced by the reduction in NDF digestibility and total VFA production. This aligns with previous findings that excessive selenium adversely affects microbial enzyme activity, leading to suppressed fermentative function and decreased VFA generation [8]. Other studies have reported similar effects of Se supplementation on VFA. Shi et al. [23] observed that a quadratic decrease in mean rumen pH and ammonia nitrogen content with increasing nanoselenium supplementation.

### 4.4. Blood Biochemical Parameters

As shown in Table 5, the CSS supplementation resulted in a reduction in TP levels in the serum of dairy buffaloes, while there was no significant effect on ALB and BUN levels. Although TP and ALB are commonly used as indicators of feed protein utilization efficiency [30], the results showed that the addition of CSS had no significant effect on milk protein and CP digestibility and facilitated an increase in milk production in buffaloes. Moreover, following the addition of CSS, the TP level was found to be within the normal range [31]. Since TP consists of ALB and globulin, this suggests that the observed reduction in TP was mainly due to the reduction in globulin. The degree of increase in globulin was associated with inflammation or liver disease [32,33]. Selenoproteins have the capacity to mitigate and repair ROS-induced liver injury, including thioredoxin reductase, glutathione peroxidase. In the present study, dairy buffalo supplemented with 0.2 mg/kg CSS exhibited significantly enhanced antioxidant capacity compared with the control group, as evidenced by higher GSH-Px activity (482 vs. 347 U/mL), along with a marked reduction in lipid peroxidation indicated by lower MDA levels (2.39 vs. 4.11 nmol/mL). These results clearly demonstrate the beneficial role of CSS in strengthening antioxidant defenses and alleviating oxidative stress. Normalizing TP and globulin levels contributes to efficient immune function, reduces the risk of chronic inflammation and immune damage, and maintains good function of organs such as the liver, as well as stable tissue osmolality, which has long-term positive effects on buffalo health in a number of ways. Of course, lower TP levels in milk production also mean that managers should pay more attention to dietary protein levels and improve feed formulations in line with milk yield. In addition, the Se status and Se intake have been negatively correlated with hepatitis, cirrhosis, and hepatocellular carcinoma [28,33]. In the present study, dairy buffaloes supplemented with 0.1 mg/kg of CSS had lower ALT and AST levels compared to the control group. Therefore, the CSS supplementation has the potential to reduce the risk of liver injury by increasing the antioxidant capacity of dairy buffaloes. TC and TG showed no statistically significant changes with Se supplementation and remained within the normal range, indicating that the buffalo lipid metabolism is in a relatively stable state.

Except for TP, CSS did not significantly affect blood biochemical parameters. Mudgal et al. [34] also obtained similar results in male buffalo calves. The supplementation of 0.3 ppm Se and other minerals (10.0 mg/kg Cu or 40 mg/kg Zn) to a high Se diet (Se: 0.28 mg/kg) had no effect on the blood metabolites of buffalo calves, probably because Se in the basal ration was already available to meet most of the nutritional requirements and the quantity of Se required to be added was minimal.

### 4.5. Antioxidant Parameters

The GSH-Px, SOD, and CAT are typically indicative of the Se status and antioxidant capacity of dairy buffaloes, while MDA levels directly reflect the level of peroxidation of the cytoplasmic membrane, which indirectly reflects the degree of cell damage. The blood glutathione peroxidase and total antioxidant capacity of dairy buffaloes with low (0.10 mg/kg) and high (0.20 mg/kg) doses of Se were higher than those of the control group (*p* < 0.05); the MDA content demonstrated a gradual decrease with the increase of Se intake. One of the primary functions of Se is to counteract oxidative stress. Its main form, selenoprotein, is involved in the Se-dependent synthesis of GSH-Px, which scavenges free radicals such as ROS, thereby protecting cells from oxidative damage [35]. The addition of Se to the diet is usually manifested as an increase in blood GPx activity. In addition, compared with the control group, the somatic cell count in the CSS group’s milk did not decrease significantly, as the count was less than 20 × 10^4^ units, indicating that the udders of dairy buffaloes were healthy. This physiological state likely limited the efficacy of CSS in further reducing somatic cell numbers. Li et al. [36] demonstrated that milk production and T-AOC and GSH-Px in dairy cows increased with dietary supplementation of hydroxy-selenomethionine. Čobanová et al. [7] reported that as Se levels increased, rumen microbes and duodenal Se concentration and mucosal GSH-Px activity increased, resulting in improved nutrient digestion and animal performance. In conclusion, the findings demonstrated that the addition of CSS has been observed to enhance antioxidant parameters in buffalo. The improvement in antioxidant parameters is likely to be directly related to the increase in milk production by protecting mammary cells from oxidative stress damage. At the same time, the improvement in antioxidant parameters also improves the overall health of the buffalo, optimizes the rumen microbial environment, and thus indirectly increases milk production.

### 4.6. Limitations of the Study

The experimental period was only 60 days, lacking monitoring of the long-term effects of CSS supplementation (e.g., throughout the entire lactation period). Short-term data showed that 0.1 mg/kg CSS increased milk production, but it remains unclear whether long-term supplementation could lead to selenium accumulation in the body, interfere with other mineral metabolism (e.g., copper and zinc), or affect reproductive performance. Future studies should be designed as long-term trials spanning the entire lactation period or even multiple parities. The research should systematically monitor the deposition patterns and dynamics of selenium and other relevant minerals in various tissues (e.g., liver, kidney, muscle, milk) and evaluate their impact on buffalo reproductive performance (e.g., postpartum estrus interval, conception rate, embryo survival).

## 5. Conclusions

The addition of 0.1 mg/kg of CSS to the diet increased milk yield and FCM of dairy buffaloes and promoted NDF digestion and total VFA production. Follow-up studies could be conducted on buffaloes at different physiological stages.

## Figures and Tables

**Table 1:** Ingredients and chemical compositions of the experimental diets.

Item	Content
Ingredient (% of DM)	
Corn silage	41.4
Fermented feed ^1^	30.0
Elephant grass	25.9
Wheat bran	2.5
Calcium phosphate	0.10
Salt	0.05
Premix ^2^	0.05
Chemical composition (% of DM)	
CP	14.78
EE	6.74
NDF	44.27
ADF	22.81
Ca	0.68
P	0.42
Se (mg/kg)	0.10

DM = dry matter; CP = crude protein; EE = ether extract; NDF = neutral detergent fiber; ADF = acid detergent fiber. ^1^ Brewer’s grain: Sweet honey feed: Granulated bean skin = 4:1:1; Sweet honey feed, provided by Nanning Sweet Honey Feed Co., Ltd. (Nanning, China), the total viable bacteria number of probiotics ≥ 1 × 10^10^ cfu/kg, the main components include *Saccharomyces cerevicae*, *Bacillus subtilis*, *Lactobacillus plantarum*, cellulase, amylase, palm meal, molasses and so on. ^2^ Premix provided the following per kg of the diet: 50 mg Fe, 10 mg Cu, 40 mg Mn, 40 mg Zn, 0.5 mg I, 0.1 mg Co, 2200 IU vitamin A, 300 IU vitamin D and 40 IU vitamin E.

**Table 2:** Effects of CSS addition on lactation performance in dairy buffaloes.

Item	Treatments ^1^				SEM	*p*-Value		
	Con	LCSS	MCSS	HCSS		Treatment	Time	Treatment × Time
Dry matter intake (kg/d)	9.35 ^c^	10.3 ^a^	10.1 ^b^	9.99 ^b^	0.39	0.001	0.584	0.767
Milk production (kg/d)								
Actual	5.78 ^b^	6.90 ^a^	6.51 ^a^	6.00 ^b^	0.85	0.001	0.019	0.997
FCM	7.24 ^d^	9.38 ^a^	8.65 ^b^	8.02 ^c^	0.11	0.001	0.440	0.970
Fat	0.328 ^d^	0.441 ^a^	0.403 ^b^	0.375 ^c^	0.06	0.001	0.898	0.983
True protein	0.240 ^b^	0.291 ^a^	0.264 ^a^	0.254 ^b^	0.04	0.001	0.467	0.995
Lactose	0.265 ^b^	0.330 ^a^	0.270 ^b^	0.248 ^b^	0.07	0.001	0.765	0.999
Milk composition (%)								
Fat	5.73 ^b^	6.46 ^a^	6.23 ^a^	6.26 ^a^	0.82	0.020	0.247	0.999
True protein	4.17	4.24	4.07	4.23	0.37	0.342	0.030	0.990
Lactose	4.61 ^a^	4.74 ^a^	4.13 ^b^	4.13 ^b^	0.71	0.003	0.485	0.998
Total solid	14.5 ^b^	15.4 ^a^	14.4 ^b^	14.6 ^b^	0.12	0.013	0.028	0.998
Somatic cell count (×10^4^ cells/mL)	17.5	15.8	16.2	15.1	0.77	0.772	0.873	0.978
Milk urea nitrogen (mg/dL)	22.4	21.2	22.7	22.8	0.28	0.159	0.043	0.290
Feed conversion ratio (kg/kg)	0.603 ^b^	0.678 ^a^	0.646 ^a^	0.602 ^b^	0.08	0.001	0.068	0.931

^a, b, c, d^ Means in the same row with different superscripts differ (*p* < 0.05). FCM = fat-corrected milk. ^1^ Control (Con) = without CSS addition; low CSS (LCSS), medium CSS (MCSS) and high CSS (HCSS) with 0.1, 0.15 and 0.2 mg Se/kg DM from CSS, respectively.

**Table 3:** Effects of CSS addition on digestibility in dairy buffaloes.

Item	Treatments ^1^				SEM	*p*-Value
	Con	LCSS	MCSS	HCSS		
DM	64.0	65.8	64.5	64.1	0.48	0.543
OM	64.8	65.9	65.1	64.5	0.41	0.685
CP	74.8	76.8	75.5	74.7	0.56	0.557
EE	61.4	65.1	64.6	63.2	1.22	0.741
Starch	93.8	94.6	93.9	93.9	0.27	0.738
NDF	56.1 ^b^	59.7 ^a^	57.7 ^ab^	57.5 ^ab^	0.39	0.003
ADF	34.1	37.6	34.1	35.8	0.59	0.087

^a, b^ Means in the same row with different superscripts differ (*p* < 0.05). DM = dry matter; OM = organic matter; CP = crude protein; EE = ether extract; NDF = neutral detergent fiber; ADF = acid detergent fiber. ^1^ Control (Con) = without CSS addition; low CSS (LCSS), medium CSS (MCSS) and high CSS (HCSS) with 0.1, 0.15 and 0.2 mg Se/kg DM from CSS, respectively.

**Table 4:** Effects of CSS addition on rumen fermentation in dairy buffaloes.

Item	Treatments ^1^				SEM	*p*-Value
	Con	LCSS	MCSS	HCSS		
pH	6.65 ^b^	6.54 ^b^	6.78 ^ab^	7.14 ^a^	0.13	0.001
Ammonia N (mg/dL)	16.1 ^a^	12.6 ^b^	12.8 ^b^	13.2 ^b^	0.46	0.035
Acetate (mmol/L)	56.56 ^b^	61.74 ^a^	59.89 ^ab^	57.60 ^b^	0.73	0.030
Propionate (mmol/L)	21.04 ^b^	23.82 ^a^	23.31 ^a^	20.37 ^b^	0.45	0.002
Isobutyrate (mmol/L)	0.96 ^ab^	1.12 ^a^	0.80 ^b^	0.74 ^b^	0.05	0.004
Butyrate (mmol/L)	8.70	7.86	8.31	6.84	0.38	0.364
Isovalerate (mmol/L)	1.60	1.74	1.40	1.27	0.08	0.109
Valerate (mmol/L)	1.26	1.38	1.21	1.02	0.06	0.184
Total VFA (mmol/L)	90.13 ^bc^	97.66 ^a^	94.92 ^ab^	87.84 ^c^	1.34	0.018

^a, b, c^ Means in the same row with different superscripts differ (*p* < 0.05). Ammonia N = ammonia nitrogen; VFA = volatile fatty acid. ^1^ Control (Con) = without CSS addition; low CSS (LCSS), medium CSS (MCSS) and high CSS (HCSS) with 0.1, 0.15 and 0.2 mg Se/kg DM from CSS, respectively.

**Table 5:** Effects of CSS addition on blood biochemical parameters in dairy buffaloes.

Item	Treatments ^1^				SEM	*p*-Value
	Con	LCSS	MCSS	HCSS		
TP (g/L)	88.4 ^a^	71.4 ^b^	70.5 ^b^	69.2 ^b^	2.46	0.001
ALB (g/L)	48.5	51.8	47.3	46.2	1.05	0.258
BUN (mmol/L)	3.48	3.96	3.88	4.57	0.19	0.766
TC (mmol/L)	2.45	2.48	2.42	3.07	0.13	0.335
TG (mmol/L)	0.347	0.307	0.314	0.365	0.13	0.498
ALT (U/L)	39.2	33.4	37.1	39.5	1.62	0.552
AST (U/L)	113	104	90.8	99.9	3.58	0.298

^a, b^ Means in the same row with different superscripts differ (*p* < 0.05). TP = total protein; ALB = albumin; BUN = blood urea nitrogen; TC = total cholesterol; TG = triglyceride; ALT = alanine aminotransferase; AST = aspartate aminotransferase. ^1^ Control (Con) = without CSS addition; low CSS (LCSS), medium CSS (MCSS) and high CSS (HCSS) with 0.1, 0.15 and 0.2 mg Se/kg DM from CSS, respectively.

**Table 6:** Effects of CSS addition on antioxidant parameters in dairy buffaloes.

Item	Treatments ^1^				SEM	*p*-Value
	Con	LCSS	MCSS	HCSS		
CAT (U/mL)	5.94	6.04	6.23	6.14	0.223	0.980
SOD (U/mL)	40.6	40.9	44.6	44.7	5.83	0.696
GSH-Px (U/mL)	347 ^b^	452 ^a^	359 ^b^	482 ^a^	19.4	0.011
MDA (nmol/mL)	4.11 ^a^	3.11 ^ab^	2.82 ^b^	2.39 ^b^	0.224	0.043
T-AOC (mM)	0.487 ^b^	0.558 ^a^	0.501 ^b^	0.687 ^a^	0.021	0.002

^a, b^ Means in the same row with different superscripts differ (*p* < 0.05). CAT = catalase; SOD = superoxide dismutase; GSH-Px = glutathione peroxidase; MDA = malondialdehyde; T-AOC = total antioxidant capacity. ^1^ Control (Con) = without CSS addition; low CSS (LCSS), medium CSS (MCSS) and high CSS (HCSS) with 0.1, 0.15 and 0.2 mg Se/kg DM from CSS, respectively.

## Data Availability

The original contributions presented in the study are included in the article.
